# Effects of Rice Bran Extract on the Quality and Digestive Properties of Chinese Steamed Buns

**DOI:** 10.3390/foods15071201

**Published:** 2026-04-02

**Authors:** Jing Liu, Zihan Zhang, Sichen Wang, Shiyi Lu, Haoran Fan, Hongyan Li, Jing Wang

**Affiliations:** Key Laboratory of Geriatric Nutrition and Health, Beijing Technology and Business University, Ministry of Education, Beijing 100048, China

**Keywords:** functional ingredient, steamed bun, rice bran extract, starch digestion rate

## Abstract

This study investigated the effects of rice bran extract (RBE) on the quality and digestibility of Chinese steamed buns (CSBs). RBE decreased the starch pasting properties, weakened the starch gel network structure, and reduced the storage modulus (*G*′) and loss modulus (*G*″). With the increase in RBE addition in CSBs, lightness difference (ΔL*) decreased, total color difference (ΔE*) increased, and the color of CSBs shifted from light to dark orange-red. Additionally, RBE increased the specific volume and enlarged the pore size of the CSBs. When 15% RBE was added, the CSBs had the lowest hardness and the highest springiness, indicating optimal quality. Notably, the addition of RBE significantly (*p* < 0.05) reduced the estimated glycemic index (eGI) from 90.916 ± 0.530 to 82.282 ± 0.399 at a 20% concentration, which represents a 9.5% reduction. This study provides a reference for the development of low-glycemic-index (GI) foods.

## 1. Introduction

Type 2 diabetes mellitus (T2DM) represents approximately 90–95% of clinically diagnosed diabetes cases, and is typically featured by a progressive reduction in insulin secretion, which is commonly associated with insulin resistance and metabolic syndrome [1,2,3]. Mounting evidence from large‑scale population epidemiological studies has confirmed that postprandial hyperglycemia is an independent risk factor for cardiovascular disease and significantly increase the risk of atherosclerosis, myocardial infarction, and all‑cause mortality. Its detrimental effect may even exceed that of fasting hyperglycemia, with this association being particularly pronounced in elderly populations and individuals with prediabetes [4,5]. Regarding dietary interventions, low glycemic index (GI) foods delay carbohydrate digestion and absorption, thereby aiding blood glucose control and reducing glycemic fluctuations, offering a sound dietary strategy for enhanced glycemic management [6]. An intervention study in T2DM patients demonstrated that a low-GI diet significantly lowered postprandial blood glucose area under the curve without adversely affecting lipid levels, indicating good clinical feasibility and metabolic benefits [7]. Furthermore, existing studies have demonstrated that low-GI foods and healthy dietary patterns abundant in whole grains, fruits, vegetables, nuts, and legumes are beneficial for better glycemic control, lower diabetes risk, and improved cardiovascular health [8].

Chinese steamed bun (CSB) is a traditional staple food, typically produced from wheat flour, yeast, and water via a series of processes including dough mixing, fermentation, shaping, proofing, and steaming [9]. Due to high-temperature steaming during production and high moisture content in the dough, starch granules undergo complete gelatinization, disrupting their original crystalline structure. This structural change makes them easily hydrolyzed by digestive enzymes, rapidly releasing glucose and elevating postprandial blood glucose levels. Consequently, CSBs typically exhibit a high GI [10]. High-GI diets trigger sharp postprandial blood glucose spikes and significant fluctuations, severely compromising glycemic control. Thus, traditional CSB is unsuitable for diabetic patients, making GI reduction crucial for their blood glucose management.

Currently, the main strategies to reduce the GI value of CSBs include raw material improvement and the addition of functional ingredients [9]. It has been shown that resistant starch, dietary fibre, polyphenols, and proteins can delay the digestion rate of starch in CSBs and reduce the eGI of CSBs [11,12,13]. These ingredients reduce the starch digestion rate by inhibiting α-amylase and α-glucosidase activities or limiting enzyme access to the substrate [10], thus reducing glucose release and controlling postprandial blood glucose. Some researchers have substituted wheat flour with potato starch and buckwheat flour to increase dietary fiber and resistant starch content in CSBs. This substitution forms a barrier around starch granules, thereby reducing enzyme activity and lowering the estimated glycemic index (eGI) of the CSBs [14,15]. In addition, bioactive peptides also modulated starch digestibility through multiple mechanisms. Peptides may adsorb onto starch granule surfaces or become embedded within the granules, forming a physical barrier that blocks amylase access to starch molecules.At the same time, there are interactions between peptides and starch that alter the internal structure of starch granules, making them more difficult to hydrolyze by enzymes. Peptides can also inhibit α-amylase, reducing the hydrolysis of starch [16,17].

Previous studies have demonstrated that rice bran peptides (RBPs) (a mixture of bioactive peptides derived from rice bran protein by enzymatic hydrolysis) exhibited a range of physiological activities, including free radical scavenging, cancer cell proliferation inhibition, blood glucose regulation, and blood pressure reduction, which contributes to the prevention and management of chronic diseases [18,19]. The same batch of rice bran extract (RBE, IC_50_ = 3.45 ± 0.17 mg/mL), the composition of which has been measured in our previous work [20], was used in this study. To further explore the potential of RBE as a functional food additive, this study aimed to incorporate RBE into refined wheat flour for the production of CSBs to investigate its effects on reducing the starch digestibility and eGI value of the CSBs. Additionally, the impact of RBE on the quality of CSBs was evaluated. This work provides a novel theoretical basis and practical insights for the application of RBE in food products.

## 2. Materials and Methods

### 2.1. Materials

Wheat flour was sourced from Wudeli Flour Group Co., Ltd. (Handan, China). Defatted rice bran was acquired from Yihai Kerry Arawana Holdings Co., Ltd. (Shanghai, China). High-activity dry yeast was obtained from Angel Yeast Co., Ltd. (Chifeng, China). Pepsin (Sigma P7000, from gastric porcine mucosa), α-amylase (Sigma A3176, from porcine pancreas), and amyloglucosidase (Sigma A7095, from *Aspergillus niger*) were purchased from Sigma-Aldrich (Shanghai, China). Alcalase (138,000 U/g) was purchased from Tianjin Yinuo Biotechnology Co., Ltd. (Tianjin, China). Anhydrous sodium sulfite (Na_2_SO_3_) and anhydrous sodium acetate were purchased from Shanghai Macklin Biochemical Technology Co., Ltd. (Shanghai, China). Hydrochloric acid (HCl) was purchased from Sinopharm Chemical Reagent Co., Ltd. (Beijing, China). Sodium hydroxide (NaOH), ethanol and glacial acetic acid were purchased from Beijing Mreda Technology Co., Ltd. (Beijing, China). All chemicals are analytical grade.

### 2.2. Preparation of RBE

The RBE used in this study was prepared according to the optimized method described in previous work [20]. Briefly, defatted rice bran was suspended in distilled water (1:5, *w*/*v*) and ground with a colloid mill for 10 min. The suspension was first incubated at 45 °C for 1.5 h in the presence of 0.02% Na_2_SO_3_ (*w*/*w*, based on the dry weight of defatted rice bran). Then, the enzymatic hydrolysis was performed at 50 °C and pH 9.5 for 4 h using alkaline protease (1000 U/g of defatted rice bran, dry weight basis). After the hydrolysis, the mixture was centrifuged (8000 rpm, 10 min). The supernatant was vacuum-concentrated and lyophilized to yield the RBE powder.

### 2.3. Pasting Properties

Pasting properties were assessed using a Rapid Visco Analyzer (RVA-TECMASTER, Perten Instruments, Beijing, China). Specifically, wheat flour samples equivalent to 3 g starch were pre-blended with RBE incorporation rates ranging from 0% to 20% (*w*/*w*, flour basis), and each blend was dispersed in 25 mL of deionized water to form a uniform suspension. The suspension was then subjected to the RVA test following a defined time-temperature profile: equilibration at 50 °C for 1 min, heating to 95 °C within 4 min, holding at 95 °C for 2 min, cooling to 50 °C over 4 min, and a final hold at 50 °C for 2 min. The stirring protocol consisted of an initial 10 s at 960 rpm, followed by 160 rpm for the remainder of the test. During the analysis, key viscosity parameters were recorded, including peak viscosity (PV), trough viscosity (TV), and final viscosity (FV). Measurements were performed in triplicate. Additionally, values for breakdown (BD) and setback (SB) were calculated using the equations described below:

(1) BD=PV−TV

(2) SB=FV−TV

### 2.4. Rheological Properties of Dough

RBE was blended with wheat flour at addition levels of 0%, 5%, 10%, 15%, and 20% (*w*/*w* of total mixture, 50 g total weight) in 30 mL distilled water until homogeneous. The blends were kneaded for 6 min with a bread maker (DL-TM018, Guangdong Donlim Electric Co., Ltd., Foshan, China) and then rested for 20 min. A suitable portion of the resulting dough was placed on the rheometer platform (DHR-1, TA Instruments, New Castle, DE, USA), and excess dough was trimmed off. A probe with a diameter of 2.5 cm was used, and the gap between the probe and platform was adjusted to 1000 μm. Dynamic strain sweep was applied to characterize the linear viscoelastic region (LVR) of the model dough. The test was performed at a strain range from 0.01% to 10%, an angular frequency of 10 rad/s, and a temperature of 25 °C, with storage modulus (*G*′) and loss modulus (*G*″) recorded. At a strain of 0.1% (within the determined LVR) and 25 °C, frequency scanning was conducted over the range of 0.1 to 100 rad/s. Rheological measurements were performed in triplicate. The *G*′ was fitted to the power-law model shown in Equation (3) [21,22], and the complex modulus (|G*|) was further calculated according to Equation (4).

(3) $$G^{\prime }=K\omega^{n}$$

(4) $$|G^{*}|=\sqrt{{\left(G^{\prime }\right)}^{2}+{\left(G^{\prime \prime }\right)}^{2}}$$

where *ω* is angular frequency, n reflects the type of intermolecular interaction, and *K* reflects the strength of intermolecular interaction.

### 2.5. Preparation of CSBs

Dry yeast (1.0 g) was dissolved in 30 mL of warm water at 37 °C to prepare the yeast solution. Dough was prepared by kneading a dry mixture of wheat flour and RBE (50 g total, with RBE replacing wheat flour at 0%, 5%, 10%, 15%, and 20%, *w*/*w*) with 30 mL of yeast solution for 6 min using a bread maker (DL-TM018, Guangdong Donlim Electric Co., Ltd., Foshan, China). The dough was then rolled through a dough sheeter with a gap of 0.5 cm, passing the dough from top to bottom through the rollers 10 times to expel air. The dough was hand-kneaded until it formed a cohesive mass and then proofed in a fermentation chamber (SPX-250B-Z, Shanghai Yonah Industrial Co., Ltd., Shanghai, China) at 30 ± 1 °C and 80–90% relative humidity for 30 min. The proofed bun dough was steamed for 25 min. The CSBs were removed from the steamer, covered with cheesecloth, and allowed to cool for 60 min before further analysis.

### 2.6. Structure and Color

After cooling for 60 min, the CSBs were evenly cut into 1.5-cm-thick slices for visual assessment and color measurement. Photographs of the sides and cross sections were taken, and the color was evaluated using a colorimeter (CM-600d, Konica Minolta, Sydney, Australia). Measurements were performed in triplicate. The color differences were calculated as follows:

(5) $$\Delta L^{*}=L_{1}^{*}-L_{0}^{*}$$

(6) $$\Delta a^{*}=a_{1}^{*}-a_{0}^{*}$$

(7) $$\Delta b^{*}=b_{1}^{*}-b_{0}^{*}$$

(8) $$\Delta E^{*}=\sqrt{{\left(\Delta L^{*}\right)}^{2}+{\left(\Delta a^{*}\right)}^{2}+{\left(\Delta b^{*}\right)}^{2}}$$

where $L_{1}^{*}$, $a_{1}^{*}$, $b_{1}^{*}$ are the lightness, red–green value, and yellow–blue value of CSBs containing RBE, respectively; $L_{0}^{*}$, $a_{0}^{*}$, $b_{0}^{*}$ are lightness, red–green value, and yellow–blue value of white CSBs; ΔL* represents lightness difference; Δa* represents red–green value difference; Δb* represents yellow–blue value difference; ΔE* represents total color difference.

### 2.7. Specific Volume

The specific volume of CSBs was determined with appropriate modifications based on the method reported by Zhang et al. [23]. After the CSBs were cooled for 60 min, each sample was weighed and its volume was measured using the rapeseed displacement method; the specific volume was then calculated as the ratio of the measured volume to the sample mass. Specific volume measurements were performed in triplicate.

### 2.8. Moisture Content

After cooling for 60 min, CSBs were homogenized with a high-speed homogenizer for 3 min. Moisture content was determined using a WG9220A forced air drying oven (Wuhan Geter Electromechanical Equipment Co., Ltd., Wuhan, China). Approximately 2–3 g of the homogenized sample was placed in a pre-weighed aluminum dish and dried at 105 °C until constant weight was achieved. Measurements were performed in triplicate.

### 2.9. pH

A 50 g sample of the homogenized CSBs (from Section 2.8) was mixed with 150 mL of distilled water and homogenized into a slurry. The pH was measured using a calibrated pH meter (PB-10, Beijing Zhongxing Weiye Instrument Co., Ltd., Beijing, China). Measurements were performed in triplicate.

### 2.10. Texture Profile

Textural properties were analyzed using a texture analyzer (CT3-25K, TA Instruments, New Castle, DE, USA) following the method of Zhang et al. [23] with modifications. After cooling at room temperature for 60 min, CSBs were sliced into uniform 1.5-cm-thick sections. Measurements were performed on the central portion of the slices. The test was conducted in TPA mode with a P/25 probe under the following settings: test speed, 1.0 mm/s; compression strain, 50%; trigger force, 5.0 g; and two compression cycles with a 10 s interval between cycles. Measurements were performed in triplicate.

### 2.11. In Vitro Digestion

Starch digestibility was assessed with slight modifications based on the method described by Zou et al. [24]. A sample containing 90 mg of starch was suspended in 6 mL of deionized water preheated to 37 °C. After 5 min of incubation, 5 mL of a 1 mg/mL pepsin solution (dissolved in 0.2 M HCl) was added to the suspension, and the mixture was continuously stirred at 37 °C for 30 min. After this step, 5 mL of 0.02 M NaOH was added to adjust the system. Next, 5 mL of a mixed digestive enzyme solution (comprising 135.26 U of porcine α-amylase and 1.23 U of amyloglucosidase in acetate buffer with pH 6.0) was incorporated, followed by incubation at 37 °C for 4 h. Aliquots of 0.1 mL were taken at 0, 5, 10, 15, 20, 30, 60, 90, 120, 180, and 240 min and promptly mixed with 0.9 mL absolute ethanol to halt the enzymatic reaction. The obtained mixture was subjected to centrifugation at 4 °C and 15,000 rpm for 10 min, and the supernatant was harvested for further analysis. The glucose concentration in the collected supernatant was quantified with a glucose oxidase-peroxidase (GOPOD) kit, and the absorbance was measured at 510 nm with a microplate reader (Synergy H1, Thermo Fisher Scientific, Shanghai, China). In vitro digestion experiments were performed in triplicate. The amount of glucose released at each time point was calculated, and starch digestibility was further computed according to the following formula:

(9) $$\%Digested=\Delta A\left(Sample\right)\times \frac{100\;µL\times 1.0\;mg/mL}{\Delta A\left(ᴅ-Glucose\;Standard\right)}\times 10\times 210\times \frac{100\%}{90\;mg}\times 0.9$$

where Δ*A*(Sample) is the difference between the absorbance of the sample and the blank; Δ*A*(d-Glucose Standard) is the difference between the absorbance of the standard and the blank; the factor 10 corrects for the dilution upon adding the aliquot to ethanol, and 210 is the dilution factor from the aliquot volume (0.1 mL) to the total reaction volume (21.0 mL); and 0.9 is the conversion factor between d-glucose and starch.

### 2.12. Fitting to First-Order Kinetics

The digestion kinetics were first analyzed using the logarithm of slope (LOS) method to identify if there were multiple digestion phases, and subsequently fitted to the combined parallel and sequential (CPS) model [25,26]. Data regarding starch digestion were fitted to Equation (10):

(10) $$C_{t}=C_{0}-\left(C_{\infty }-C_{0}\right)\times \left(1-e^{-kt}\right)$$

where *C_t_* and *C*_0_ denote the starch digestibility at time *t* min and 0 min, *C*_∞_ is the estimated percentage of starch digested at the end point of the reaction, and *k* stands for the starch digestion rate coefficient. A linear relationship exists between ln(*dC_t_*/*dt*) and *k*. Therefore, the above equation can be further transformed into the following:

(11) $$\ln\left(dC_{t}/dt\right)=-kt+\ln\left(C_{\infty }-C_{0}\right)$$

The CPS model was used to fit the digestion profiles and quantify the kinetic parameters. The model is defined by the following equations:

(12) $$C_{t}=C_{0}+\left(C_{1\infty }\right)\times \left(1-e^{-k_{1}t}\right)+\{\begin{array}{c} \left(C_{2\infty }\right)\times \left(1-e^{-k_{2}\left(t-t_{2start}\right)}\right),t\geq t_{2start}\\ 0,\;t<t_{2start} \end{array}$$

*C*_0_ denotes the starch digestibility at time 0, *C*_1∞_ and *C*_2∞_ denote the ultimate starch digestibility of digestible fraction 1 and digestible fraction 2, *t*_2start_ denotes the time when slow digestion starts, *k*_1_ and *k*_2_ denote the fast and slow digestion rate coefficient, respectively.

### 2.13. eGI

Based on the in vitro digestion kinetic curves, the area under the starch digestibility curve (AUC) was calculated for both CSBs and white bread control within 0–120 min. The hydrolysis index (HI) was then determined using Equation (13), followed by calculation of the eGI using Equation (14) [27].

(13) $$HI=\frac{S\left(Sample\right)}{S\left(White\;bread\right)}\times 100$$

(14) eGI=(0.549×HI)+39.71

where *S*(Sample) and *S*(White bread) denote the respective areas under the digestion curves for the sample and white bread.

### 2.14. Statistical Analysis

All experimental measurements in the present study were reported as the mean ± standard deviation. The significance of statistical differences was analyzed by means of one-way analysis of variance (ANOVA) with the aid of SPSS 26.0 software (SPSS Inc., Chicago, IL, USA, 2009). Statistical significance was defined as a *p*-value lower than 0.05. All experimental figures were plotted using Origin 2018 software (Origin Lab Corporation, Northampton, MA, USA).

## 3. Results and Discussion

### 3.1. Effects of RBE on Wheat Dough Properties

#### 3.1.1. Pasting Properties Analysis

**Figure 1:**
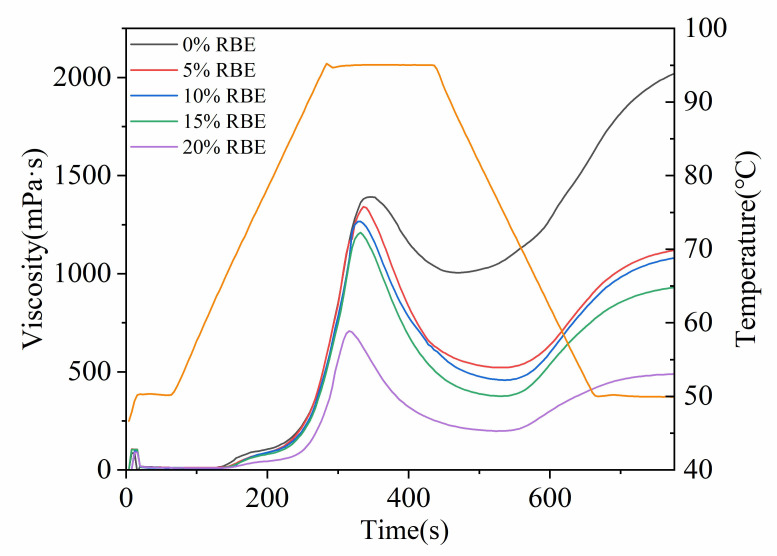
The effects of RBE on starch pasting properties. The yellow line represents temperature.

The pasting properties of the RBE-wheat flour mixture are shown in Figure 1 (pasting curve) and Table S1 (pasting parameters). As the RBE addition increased, PV, TV, and FV decreased accordingly. When RBE was added at 20%, PV, TV, and FV decreased by 48.39%, 80.00%, and 75.41%, respectively, reaching the lowest levels (*p* < 0.05). This was partly due to RBE dilution of starch concentration in the system and partly because RBE limited starch water absorption and swelling by competing for water and forming a physical barrier [28,29]. As shown in Figure 1, RBE addition shifted the peak time to the left and reduced peak viscosity, indicating that RBE accelerated the gelatinization process but limited the degree of gelatinization. This resulted in a weaker gel network, diminished thermal stability of the starch, and increased susceptibility to rupture under high-temperature shear. The BD values in all RBE-added groups were significantly higher than those in the blank group. However, the BD value of the 20% RBE group was lower than that of groups with lower RBE addition levels, which can be attributed to its extremely low PV. The SB value in the 20% RBE group decreased by 70.87% (*p* < 0.05) compared to the control group. This indicated that RBE weakens the hydrogen bonds between amylose and amylopectin starch during leaching, significantly suppressing molecular rearrangement and short-term retrogradation during cooling, thereby enhancing the cold stability of starch [30].

#### 3.1.2. Rheological Properties

**Figure 2:**
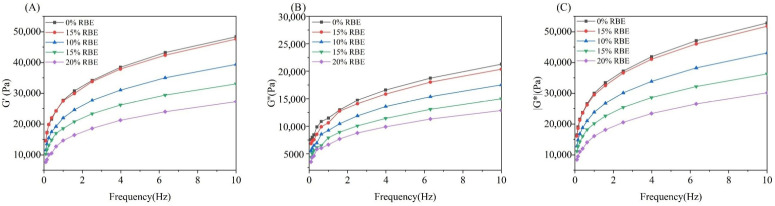
The effects of RBE on dough rheological properties, (**A**) storage modulus (*G*′), (**B**) loss modulus (*G*″), and (**C**) complex shear modulus (|G*|).

The rheological properties of the dough samples supplemented with RBE are presented in Figure 2 and Table 1. In the range of 0.1 Hz–10 Hz, *G*′, *G*″, and |G*| of all doughs increased with frequency, and all doughs had *G*′ > *G*″, indicating that all samples exhibited typical elastic solid-like behavior [23]. *G*′, *G*″, and |G*| decreased with increasing RBE addition, indicating that RBE weakened the overall viscoelasticity of the dough. This observation could be attributed to the dilution of gluten proteins and the weakening of the starch gel network by RBE [28]. Previous studies have shown that peptides could attenuate the electrostatic repulsion between starch chains and reduce the chain conformation, which in turn reduces the density and order of the gel structure and weakens the gel network [31].

The results from the power-law model fitting (Table 1) indicate that the coefficient of determination (R^2^) for all samples exceeded 0.988, demonstrating the high reliability of the model fit. The *n* value reflected the dependence of *G*′ on frequency, while the *K* value indicates dough strength. As shown in Table 1, the *n* values for all doughs ranged from 0 to 1, exhibiting typical weak gel characteristics. At the 20% RBE addition level, the *n* value significantly increased, indicating that *G*′ became more dependent on frequency and the stability of the dough’s network structure decreased. This suggests that a high RBE addition (20%) weakened the dough’s network structure, making it more susceptible to breakdown under rapid deformation. The *K* value decreased significantly with increasing RBE addition. When the addition was ≤5%, the *K* value exhibited no significant difference compared with the control group. However, at 10% and 15% addition levels, the *K* value decreased significantly by approximately 21.11% and 31.73%, respectively. A 49.76% reduction in the *K* value was observed at the 20% RBE addition level. This decline results from the dilution of gluten proteins by RBE and its physical inhibition of gluten network formation, which led to diminished dough structural integrity [21].

### 3.2. Effect of RBE on the Quality of CSBs

#### 3.2.1. Structure and Color Analysis

**Figure 3:**
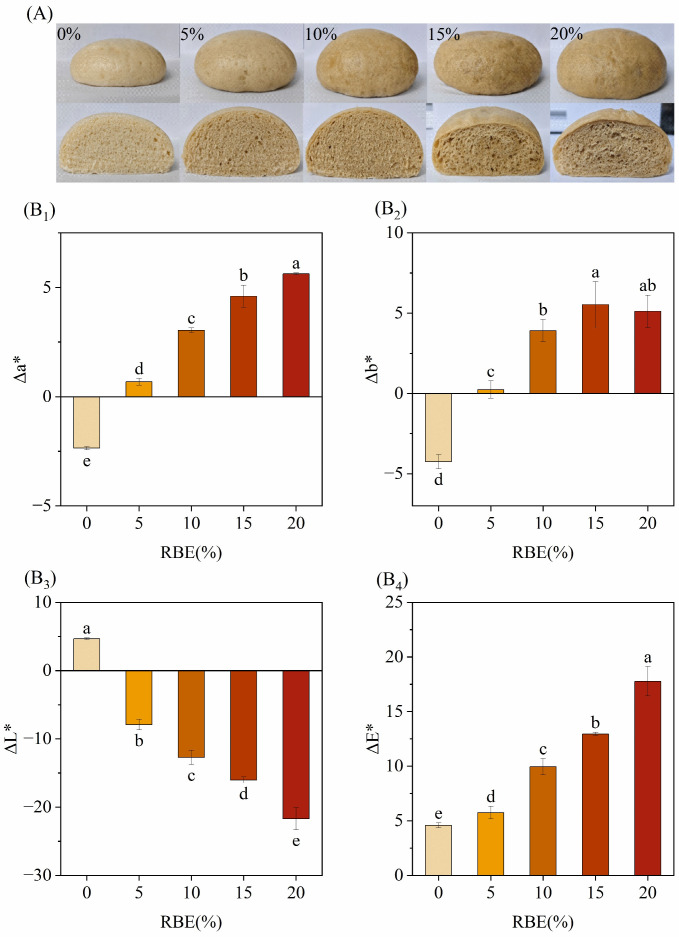
Effects of RBE on the texture and color of CSBs. (**A**) The appearance of CSBs with different additions of RBE; (**B_1_**) The effects of rice bran extract (RBE) on the red–green value difference (Δa*) of Chinese steamed buns (CSBs); (**B_2_**) The effects of rice bran extract (RBE) on the yellow–blue value difference (Δb*) of Chinese steamed buns (CSBs); (**B_3_**) The effects of rice bran extract (RBE) on the lightness difference (ΔL*) of Chinese steamed buns (CSBs); (**B_4_**) The effects of different additions on the total color difference (ΔE*) of Chinese steamed buns (CSBs). Values with different lowercase letters are significantly different (*p* < 0.05).

The side and cross-section of the CSBs made by mixing RBE and wheat flour are shown in Figure 3A. The internal structure of white CSBs (RBE addition of 0) was uniform, with small and dense stomata and no larger bubbles; the stomata of the CSBs gradually increased after the addition of RBE, which is likely due to RBE enhancing the yeast’s gas-producing capacity [23]. At 15% RBE, larger bubbles appeared. This phenomenon was more obvious at 20%, and the distribution of pores tended to be disordered. The CSBs with 5% and 10% RBE maintained a more uniform pore distribution compared to those with higher RBE levels.

The CSBs’ color showed regular changes (Figure 3B_1_–B_4_). With the increase in RBE addition, ΔL* decreased from 4.67 ± 0.12 to −21.72 ± 1.61, indicating that the dark substances in RBE caused a significant decrease in the lightness of the CSBs (*p* < 0.05); Δa* value continued to increase from −2.36 ± 0.08 to 5.63 ± 0.04, and Δb* value increased from −4.25 ± 0.44 to 5.12 ± 1.00, indicating that the color of the CSBs shifted gradually towards red and yellow; ΔE* gradually increased (4.58 ± 0.22 to 17.77 ± 1.34), indicating that the total color difference gradually increased and the color change became increasingly visible. These results demonstrated that the color alteration of CSBs was directly proportional to the RBE addition level, a consequence of the intrinsic pigments in RBE.

#### 3.2.2. Specific Volume, Moisture Content, and pH

**Figure 4:**
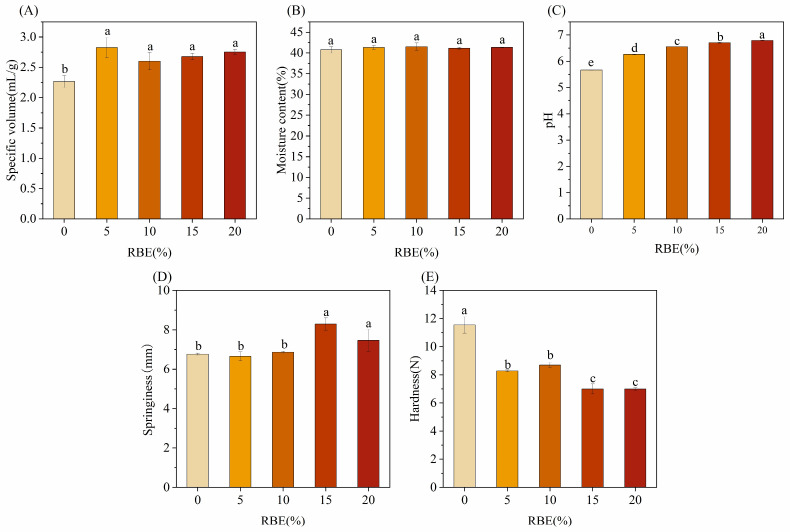
Physical attributes of CSBs. (**A**) The effects of rice bran extract (RBE) on the Specific volume of Chinese steamed buns (CSBs); (**B**) The effects of rice bran extract (RBE) on the moisture content of Chinese steamed buns (CSBs); (**C**) The effects of rice bran extract (RBE) on the pH of Chinese steamed buns (CSBs); (**D**) The effects of rice bran extract (RBE) on the springiness of Chinese steamed buns (CSBs); (**E**) The effects of rice bran extract (RBE) on the hardness of Chinese steamed buns (CSBs). Values with different lowercase letters are significantly different (*p* < 0.05).

Specific volume, moisture content, and pH served as critical physicochemical indicators for evaluating CSBs’ quality, profoundly influencing their texture, flavor, and appearance. Specific volume reflected the degree of expansion and structural looseness of CSBs, moisture content directly affected the texture and shelf life of CSBs, and pH reflected the taste and texture of buns. As shown in Figure 4A–C, the specific volume of CSBs was the largest when 5% RBE was added. This improvement is likely due to RBE enhancing CSBs’ gas retention ability [23], thereby preventing the CSBs from collapsing and resulting in a softer texture. RBE significantly increased the pH of the CSBs, an effect potentially attributable to its content of alkaline amino acids. The moisture content of all CSB samples ranged from 40.76% to 41.53%, which fell well within the typical range of 39–44% reported for CSB crumb in the literature [32]. This indicates that RBE incorporation did not adversely affect the moisture retention of the products.

#### 3.2.3. Texture Profile Analysis

The regulation of the hardness and elasticity of the CSBs by RBE addition was revealed by texture profile analysis (Figure 4D,E). Hardness decreased sharply from 11.55 ± 0.58 N to 8.28 ± 0.06 N (*p* < 0.05) at 5% of RBE addition, and no significant difference was observed between 5% and 10% RBE. A further significant decrease in hardness occurred at 15% RBE, with no significant change thereafter up to 20%, which indicated that RBE could soften the CSBs to improve the quality [33]. There was no significant difference in the springiness of RBE in the range of 0–10% (*p* > 0.05), and springiness jumped from 6.77 ± 0.06 mm to 8.3 ± 0.31 mm when the additive amount was increased to 15% (*p* < 0.05), which reached the maximum value of the experiment. The springiness fell back to 7.47 ± 0.56 mm at 20% addition, suggesting the existence of an optimal concentration threshold. Overall, RBE addition significantly improved the textural properties of CSBs by reducing hardness and enhancing springiness. The reduction in hardness observed with RBE addition was particularly noteworthy when compared with the effects of other functional ingredients reported in the literature. Previous studies have consistently shown that incorporating ingredients such as buckwheat flour or resistant starch to reduce glycemic index typically compromises textural quality, resulting in increased hardness [12,15]. In striking contrast, our results demonstrated that RBE significantly reduced hardness, with the 15% formulation achieving the lowest value among all samples.

### 3.3. Effect of RBE on the In Vitro Digestive Properties of CSBs

**Figure 5:**
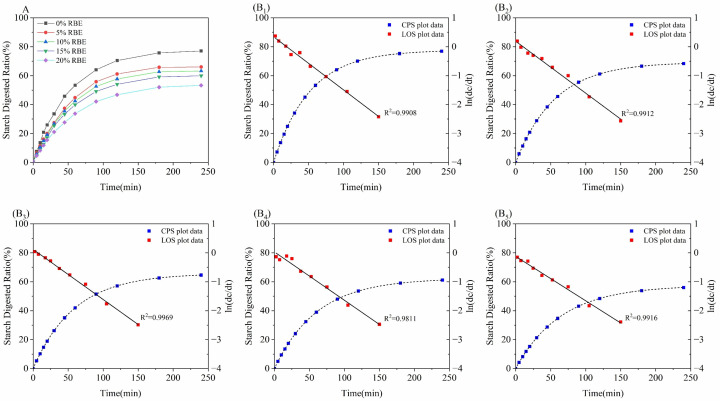
Effect of rice bran extract (RBE) on the in vitro digestive properties of Chinese steamed buns (CSBs). (**A**) Digestive curves of CSBs with different RBE additions. (**B_1_**–**B_5_**) Logarithm of slope (LOS) and combined parallel and sequential (CPS) fitted curves of digestive curves for CSBs with different RBE additions, (**B_1_**) 0%RBE; (**B_2_**) 5%RBE; (**B_3_**) 10%RBE; (**B_4_**) 15%RBE; (**B_5_**) 20%RBE.

The digestive characteristics of CSBs were determined through in vitro simulated digestion experiments. The digestion curves of the CSBs are shown in Figure 5A. The starch digestibility of all CSBs initially increased rapidly, indicating that starch was rapidly broken down during the early stages of in vitro digestion. As starch content decreased, starch digestibility gradually stabilized. The CSBs without RBE addition exhibited the fastest increase in starch digestibility and the highest overall starch digestibility. After RBE addition, the starch digestibility of CSBs significantly decreased, exhibiting a downward trend with increasing RBE content. As shown in Table 1, with RBE addition rising from 0% to 20%, corresponding eGI values declined from 90.916 ± 0.530 to 82.282 ± 0.399 (*p* < 0.05). This reduction could be attributed to the inhibitory effect of RBE on starch digestive enzymes, consistent with its α-glucosidase inhibitory activity reported previously [20], thereby slowing glucose release. This enhances the CSBs’ digestion-resistant properties, reducing both starch digestibility and eGI value. Furthermore, as reported by Chen et al. [16], peptides could adsorb onto the surface of starch granules, forming a physical barrier that limits enzyme access and reduces rapidly digestible starch content. This mechanism is consistent with the observed decrease in starch digestion rate during the initial phase of the digestion curve following the addition of RBE.

To further quantify the inhibitory effect of RBE on starch digestion, the digestion curves were fitted using the LOS and CPS models (Figure 5B_1_–B_5_). The derived kinetic parameters, including the *k* and *C_∞_*, are summarized in Table 1. Both kinetic parameters decreased significantly with higher RBE concentrations: *k* (×10^−2^) decreased from 1.933 ± 0.024 to 1.535 ± 0.005 (*p* < 0.05), and *C_∞_* decreased from 77.643 ± 0.818 to 54.877 ± 2.592 (*p* < 0.05). This decrease in kinetic parameters is consistent with the findings reported by Liu et al. [34]. This indicated that the active peptides derived from rice bran in RBE exert a retarding effect on starch hydrolysis through the competitive inhibition of α-glucosidase activity, thereby contributing to the reduced estimated glycemic index (eGI). Although the eGI values of all CSB samples remained within the high-GI category (>70), the addition of 20% RBE resulted in a statistically significant 9.5% reduction (from 90.916 ± 0.530 to 82.282 ± 0.399, *p* < 0.05). While this shift does not reclassify the product as medium or low-GI, such a reduction might still have clinical relevance. A classic study by Brand et al. [7] demonstrated that reducing dietary GI from 91 to 77 (a 15% reduction) led to an 11% decrease in HbA1c levels in individuals with type 2 diabetes, despite the overall diet GI remaining within the high-GI range. Therefore, RBE incorporation might serve as a practical strategy to improve the nutritional profile of CSBs.

## 4. Conclusions

The effects of rice bran extract (RBE) on the pasting properties of flour, rheological properties of dough, and quality and digestibility of Chinese steamed buns (CSBs) were investigated. The results showed that RBE could reduce the pasting properties of starch, weaken the starch gel network structure, and enhance the quality of CSBs. Notably, CSB with 15% RBE exhibited the lowest hardness, highest springiness, highest specific volume, and overall optimal quality. With the increase in RBE addition, the starch digestibility and the estimated glycemic index (eGI) of the CSBs decreased. The interaction between RBE and starch, along with the inhibitory effect of rice bran peptides on α-glucosidase activity, reduced the starch digestion rate coefficient and the estimated percentage of starch digested at the end point of the reaction. These findings demonstrate that RBE is a promising functional ingredient for developing CSBs with improved textural quality and a lower glycemic response. From an industrial perspective, the RBE could be produced via a cost-effective and scalable one-step enzymatic hydrolysis process [20], supporting the feasibility of its large-scale application. The optimal addition level of 15% RBE identified here provides a practical formulation for producing CSBs with enhanced texture and reduced glycemic response. Nevertheless, further research is warranted to assess the full economic viability and product stability of RBE-enriched CSBs for commercial production. Additionally, sensory evaluation was not performed in this study and should be included in future research to assess consumer acceptance. Further studies are also needed to investigate the direct effects of RBE on yeast fermentation kinetics and to elucidate the molecular mechanisms underlying the interactions of RBE with gluten proteins, which would further clarify the observed structure–function relationships. This study provides a theoretical basis for the application of RBE in reduced glycemic index staple food products.

## Figures and Tables

**Table 1:** The *n*, *K* and R^2^ values obtained by fitting the frequency sweep data into the power law model. Starch digestion rate coefficient (*k*), estimated percentage of starch digested at the end point of the reaction (*C*_∞_), and estimated glycemic index (eGI) of Chinese steamed buns (CSBs) with rice bran extract (RBE).

RBE (%)		0	5	10	15	20
Rheological properties	*n*	0.253 ± 0.009 ^b^	0.248 ± 0.012 ^b^	0.260 ± 0.006 ^ab^	0.252 ± 0.013 ^b^	0.279 ± 0.011 ^a^
	*K*	17,115.761 ± 229.702 ^a^	17,132.570 ± 87.951 ^a^	13,501.951 ± 1739.198 ^b^	11,685.588 ± 470.082 ^b^	8598.224 ± 1241.245 ^c^
	R^2^	0.988 ± 0.003	0.996 ± 0.002	0.991 ± 0.005	0.993 ± 0.003	0.995 ± 0.002
Digestive properties	*k* (×10^−2^)	1.933 ± 0.024 ^a^	1.795 ± 0.005 ^b^	1.693 ± 0.011 ^c^	1.670 ± 0.030 ^c^	1.535 ± 0.005 ^d^
	*C*_∞_ (%)	77.643 ± 0.818 ^a^	67.565 ± 0.924 ^b^	65.754 ± 1.311 ^bc^	61.215 ± 0.013 ^c^	54.877 ± 2.592 ^d^
	eGI	90.916 ± 0.530 ^a^	87.211 ± 0.976 ^b^	86.902 ± 0.769 ^bc^	86.498 ± 0.093 ^c^	82.282 ± 0.399 ^d^

Data are presented as means ± standard deviations. Values with different lowercase letters in the same row are significantly different (*p* < 0.05). *n*, the degree of dependence of *G*′ on frequency sweep; *K*, the strength of intermolecular interaction; R^2^, the coefficient of determination.

## Data Availability

The original contributions presented in this study are included in the article and Supplementary Materials. Further inquiries can be directed to the corresponding author.

## Supplementary Materials

The following supporting information can be downloaded at https://www.mdpi.com/article/10.3390/foods15071201/s1. Table S1. Pasting properties of starch with added RBE. PV, TV, FV, BD, and SB represent peak viscosity, trough viscosity, final viscosity, breakdown value, and setback value, respectively.
