# Supplementary material for: Effects of Rice Bran Extract on the Quality and Digestive Properties of Chinese Steamed Buns
Source: Foods. 2026 Apr 2;15(7):1201. doi: 10.3390/foods15071201 (PMC13073980; doi:10.3390/foods15071201)
Supplement: Supplementary file 1 [file foods-15-01201-s001.zip › foods-4188862-supplementary.pdf]

Table S1. Pasting properties of starch with added rice bran extract (RBE). PV, TV, FV, BD, and SB represent peak viscosity, trough viscosity, final viscosity, breakdown value, and setback value, respectively.

RBE(%)	PV（mPa·s）	TV（mPa·s）	FV（mPa·s）	BD（mPa·s）	SB（mPa·s）
0	1426 ± 35^{a}	1030 ± 25^{a}	2070 ± 50^{a}	396 ± 10^{c}	1040 ± 25^{a}
5	1342 ± 50^{ab}	526 ± 17^{b}	1120 ± 37^{b}	816 ± 33^{a}	594 ± 20^{b}
10	1267 ± 42^{b}	458 ± 17^{c}	1080 ± 40^{b}	809 ± 25^{a}	622 ± 23^{b}
15	1231 ± 21^{b}	383 ± 7^{d}	946 ± 16^{c}	849 ± 15^{a}	564 ± 10^{b}
20	736 ± 28^{c}	206 ± 8^{e}	509 ± 20^{d}	530 ± 20^{b}	303 ± 12^{c}

Data are presented as means ± standard deviations. Values with different lowercase letters in the same column are significantly different ($p < 0.05$).
